# Efficacy and Safety of Oral Spironolactone for Women With Acne Vulgaris: A Systematic Review and Meta‐Analysis of Randomized Placebo‐Controlled Trials With Trial Sequential Analysis

**DOI:** 10.1111/jocd.70411

**Published:** 2025-08-18

**Authors:** Laura Ghanem, Najwaa Kirmani, Nathalia De León Fernández, María Paula Palacios‐Ortiz, Juan David Rodríguez‐Parra, Corina A. Rusu

**Affiliations:** ^1^ Faculty of Medical Sciences Lebanese University Beirut Lebanon; ^2^ Dow Medical College Karachi Pakistan; ^3^ Universidad de los Andes Bogotá Colombia; ^4^ Universidad San Francisco de Quito Quito Ecuador; ^5^ Pontificia Universidad Javeriana Bogotá Colombia; ^6^ Dermatology Department University of Virginia Charlottesville Virginia USA

**Keywords:** acne vulgaris, spironolactone, women

## Abstract

**Background:**

Adult acne vulgaris is a chronic inflammatory skin condition that primarily affects females. Initial management includes topical and oral medications, but important limitations include ineffectiveness, nonadherence, and adverse effects. Spironolactone has shown good results in off‐label acne management.

**Aims:**

We aim to conduct a systematic review and meta‐analysis exploring the safety and efficacy of oral spironolactone for females with acne.

**Methods:**

We searched PubMed, Embase, and Cochrane for randomized controlled trials (RCTs) comparing oral spironolactone in women with acne to placebo. The primary endpoint was the objective assessment of acne improvement. Secondary endpoints included subjective assessment and adverse events. Statistical analysis was performed using Review Manager 5.4. Heterogeneity was assessed with *I* ^2^ statistics.

**Results:**

We included 563 patients from 5 RCTs, of which 251 (42.9%) received spironolactone. Objective assessment of acne improvement (OR 6.59; 95% 3.50–12.43; *p* < 0.00001; *I* ^2^ = 0%) was sixfold higher in the spironolactone group compared with placebo. Subjective assessment showed no difference between the two groups (OR 5.22; 95% 0.62–44.24; *p* < 0.13; *I* ^2^ = 85%). Menstrual irregularities (OR 1.09; 95% 0.37–3.25; *p* = 0.88; *I* ^2^ = 33%) and breast enlargement (OR 1.37; 95% 0.79–2.38; *p* = 0.26; *I* ^2^ = 0%) were nonsignificant in patients taking spironolactone. Trial sequential analysis (TSA) confirmed that the required sample size was reached, favoring spironolactone over placebo.

**Conclusion:**

Our study suggests that oral spironolactone improves acne in female patients compared to placebo without increasing risks; thus, it should be elevated from “off‐label” use to an officially recommended standard of care.

**PROSPERO Registration:**

CRD42024626984.

**Abbreviations**
AAD: American Academy of Dermatology
AFAST: adult female acne scoring tool
FDA: Food and Drug Administration
IGA: Investigator's Global Assessment
NICE: National Institute for Health and Care Excellence
OR: odds ratio
PCOS: polycystic ovary syndrome
PRISMA: Preferred Reporting Items for Systematic Reviews and Meta‐Analyses
PROSPERO: International Registry of Systematic Reviews (National Institute for Health Research)
RCT: randomized controlled trial (s)
SAFA: Study of the Assessment of the Effects of spironolactone on Acne
TSA: trial sequential analysis

## 1 Introduction

Acne vulgaris is a persistent, chronic, inflammatory skin condition affecting the pilosebaceous follicles [1]. It is estimated to be the eighth most prevalent disease worldwide, particularly during teenage and early adult years—which often affects females—with a prevalence that remains broadly consistent globally [2]. Despite the extensive knowledge of acne pathogenesis, new regimes of treatment and combinations continue to be the focus of discussion and research [1].

The National Institute for Health and Care Excellence (NICE) and the American Academy of Dermatology (AAD) guidelines on acne vulgaris management recommend various treatments depending on acne severity and clinical presentation [3, 4]. These include physical and chemical therapies, antibiotics, and retinoids administered either topically or via the systemic oral route [3, 4]. In adult female acne, spironolactone, an androgen receptor blocker, has demonstrated efficacy as an off‐label treatment for acne [5]. However, its recommendation in clinical guidelines is still based primarily on expert opinions or consensus.

Despite the recent publication of two randomized, controlled, double‐blinded trials evaluating the effectiveness of spironolactone in adult female acne [6, 7], along with previously available studies in the literature [8, 9, 10], an aggregate analysis synthesizing this information to comprehensively assess the effectiveness of spironolactone in acne remains unavailable.

Therefore, we aimed to conduct a systematic review and meta‐analysis with trial sequential analysis (TSA) to evaluate the efficacy and safety of oral spironolactone in women with acne vulgaris.

## 2 Methods

This Meta‐Analysis and Systematic Review was registered in the International Prospective Register of Systematic Reviews (PROSPERO) under registration number (CRD42024626984), performed under the Cochrane Handbook for Systematic Reviews of Interventions, and reported adhering to the Preferred Reporting Items for Systematic Reviews (PRISMA) statement guidelines [11].

### 2.1 Eligibility Criteria

Studies that met the following eligibility criteria were included: (1) randomized controlled trials (RCTs); (2) comparing oral spironolactone with placebo; (3) reporting the results in females; and (4) reporting at least one clinical outcome of interest. No restrictions were applied to follow‐up time. We excluded studies with: (1) overlapping patient populations; (2) observational design; or (3) without a placebo control group.

### 2.2 Search Strategy and Data Extraction

We systematically searched Embase, Cochrane Central and PubMed databases from inception to December 2024, for studies published in any language using the following search terms: “spironolactone”, “aldactone”, “Verospiron”, “Spirolone”, “SC 9420”, “mineralocorticoid receptor antagonist”, “aldosterone antagonist”, “acne”, “comedones”, “seborrhea”, “Propionibacterium acnes”, “Cutibacterium acnes”, “randomized”. Boolean operators like “OR” and “AND” were used to optimize the search results. The complete search strategy can be found in the Appendix S1. Study selection and data extraction were performed by two authors (L.G. and N.K.) independently. Data extraction for baseline study characteristics reported in Table 1 was performed by two authors (L.G. and N.K.). Outcomes data extraction was conducted by two additional authors (N.D.L. and M.P.P). Any disagreements were resolved among the research group.

**TABLE 1:** Baseline characteristics of included studies.

Study	Oral spironolactone	Concurrent topical treatment	Follow‐up (months)	No. of female patients, *n*	Age (years), range	BMI, (kg/m^2^), median (SD)	Tool of acne diagnosis	Total lesions, mean (SD)	Inflammatory lesions, mean (SD)	Acne severity	PCOS suspicion/diagnosis, *n* (%)
Patiyasikunt 2020	25 mg or 50 mg daily	Yes^e^	3	63	30.17^a^ 25–45	22.83 (4.22)	Total, comedonal, and inflammatory lesions, AFAST‐F and AFAST‐S scores	45.9 (31.21)	8.23 (6.30)	Moderate to severe	0 (0%)
Goodfellow 1984	50 mg, 100 mg, 150 mg, or 200 mg daily	No	3	26	24^a^ 18–38	N/A	Sebum secretion	N/A	N/A	Severe	N/A
Muhlemann 1986	200 mg daily	No	3	29	NR	N/A	Total and inflammatory lesions	30.5 (4.5)^c^	40.12 (4.98)^c^	Moderate to severe	N/A
Mansurul 2000	50 mg daily	No	3	56	10–40	N/A	Grading	N/A	N/A	N/A	N/A
SAFA 2024	50 mg, 100 mg^d^	Yes^e^	≤ 12	410	29.2^a^ (7.2)^b^	26.1 (5.6)	Acne‐QoL and IGA score	N/A	N/A	Mild to severe^f^	77 (19.4%)

Abbreviations: AFAST‐F, adult female acne score tool of the face; AFAST‐S, adult female acne score tool of the submandibular areas; BMI, body mass index; IGA, Investigator's Global Assessment; N/A, not applicable; NR, not reported; PCOS, polycystic ovary syndrome; QoL, quality of life.

^a^ Mean or median.

^b^ Standard deviation (SD).

^c^ Standard error of the mean (SEM).

^d^ SPL 50 mg daily for 6 weeks; then increased to 100 mg until 24 weeks.

^e^ Topical benzoyl peroxide 2.5% gel, continued to use.

^f^ Women with an Investigator's Global Assessment (IGA) ≥ 2 were eligible to participate in the trial.

### 2.3 Endpoints

The primary endpoint was improvement of acne through objective assessment. The efficacy of acne treatment is generally evaluated through different clinical scales, most of which reflect an objective view. The most used ones are the Investigator's Global Assessment (IGA), adult female acne scoring tool (AFAST), among others [12, 13]. The results gathered through these scales are typically synthesized in clinical trials in a dichotomic manner (improvement/no improvement) or trichotomic manner (improvement/no improvement/worse). For this meta‐analysis and systematic review, we considered the scales to be comparable with each other.

Secondary endpoints were subjective assessment and adverse effects such as breast enlargement and menstrual irregularities.

### 2.4 Statistical Analysis

Odds ratios (ORs) with 95% confidence intervals were used to compare the efficacy of spironolactone with placebo in acne improvement, based on the objective and subjective assessments, as well as the adverse events. Heterogeneity was assessed with *I* ^2^ statistics and the Cochran *Q* test; *p*‐values < 0.10 and *I* ^2^ > 25% were considered significant for heterogeneity. The DerSimonian and Laird random‐effects model was used. Leave‐one‐out sensitivity analyses were performed for the subjective assessment of acne improvement to ensure the results were not dependent on a single study. In addition, we generated a TSA for our primary endpoint—the objective assessment of acne improvement—to minimize the risk of Type 1 and Type 2 errors by adjusting significance thresholds and ensuring adequate statistical power or sample size. We used Review Manager 5.4 (Cochrane Center, The Cochrane Collaboration, Denmark) for statistical analysis, while TSA software was utilized for the TSA.

### 2.5 Quality Assessment

Cochrane Collaboration's tool for assessing risk of bias in randomized trials (Rob 2) was used to assess the quality of individual RCTs [14]. Two independent authors completed the risk of bias assessment (L.G. and J.D.R.). Disagreements were resolved through consensus after discussing reasons for the discrepancy.

## 3 Results

### 3.1 Study Selection and Baseline Characteristics

**FIGURE 1:**
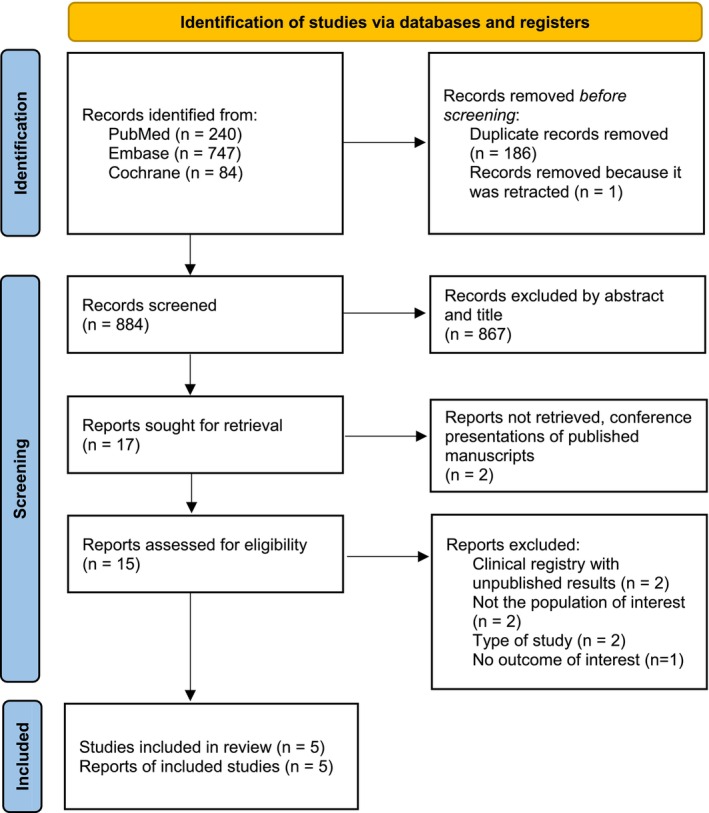
PRISMA flow diagram of study screening and selection.

The initial search yielded 1071 results. After removal of duplicate records and ineligible studies, 15 remained and were fully reviewed based on the inclusion criteria. Of these, a total of five randomized controlled trials (RCTs) were included, comprising 563 patients (Figure 1). A total of 251 (44.6%) women received spironolactone; study characteristics are reported in Table 1. Two studies included concurrent topical acne treatment [6, 7]; the remaining studies all used spironolactone exclusively as part of acne treatment. There were variations among studies regarding the follow‐up periods, the acne vulgaris severity assessment, and the dosage of spironolactone (Table 1).

### 3.2 Pooled Analysis of All Studies

**FIGURE 2:**
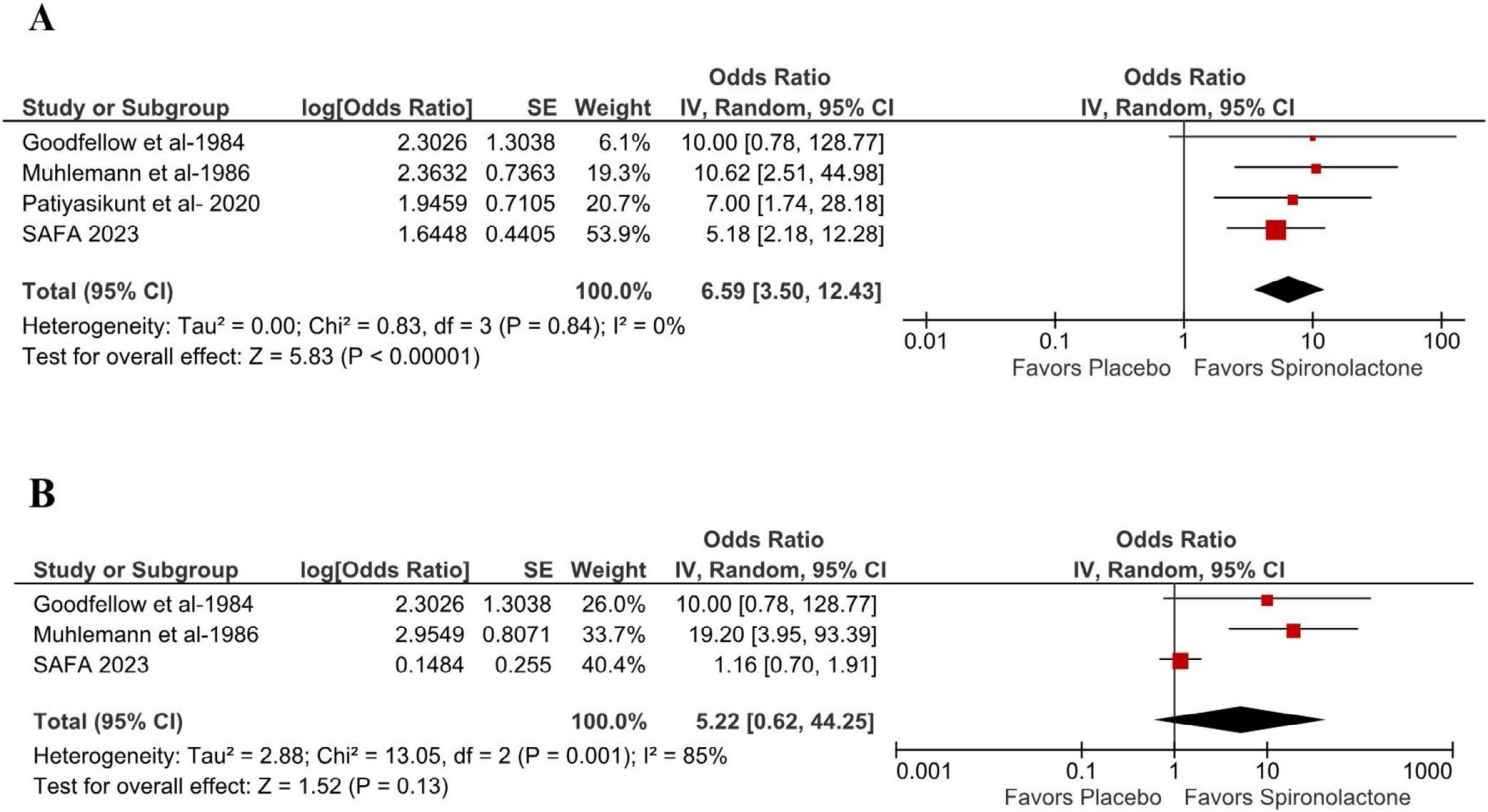
The severity of acne vulgaris was significantly reduced in women taking spironolactone as compared to placebo, following the objective and subjective assessment. CI, confidence interval; IV, inverse variance; OR, odds ratio; SE, standard error.

The primary endpoint analyzed was the objective assessment using IGA or other scales of acne improvement, which was sixfold higher in those receiving spironolactone as compared with placebo (OR 6.59; 95% 3.50–12.43; *p* < 0.00001; *I* ^2^ = 0%; Figure 2A).

However, subjective assessment showed no difference between the two groups (OR 5.22; 95% 0.62–44.25; *p* = 0.13; *I* ^2^ = 85%; Figure 2B). The improvement of acne vulgaris was significantly noted in women taking spironolactone as compared to placebo when unadjusted odd estimates were used, aligning with results of adjusted odd estimates in both objective and subjective assessment (Figures S1 and S2).

**FIGURE 3:**
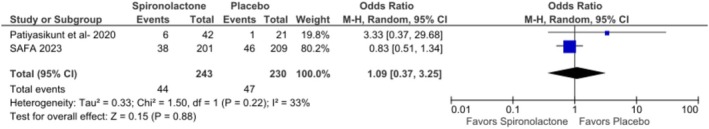
The severity of menstrual irregularities is not significantly seen in women taking spironolactone for acne vulgaris up to 12 weeks. CI, confidence interval.

**FIGURE 4:**
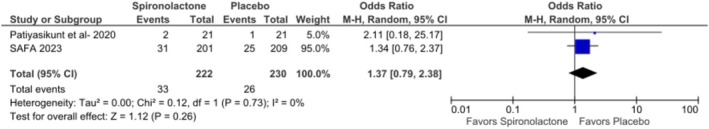
The severity of breast enlargement is higher in women taking spironolactone for acne vulgaris compared with placebo. CI, confidence interval.

Rates of menstrual irregularities (18.1% vs. 20.4%; OR 1.09; 95% 0.37–3.25; *p* = 0.88; *I* ^2^ = 33%; Figure 3) and breast enlargement (14.9% vs. 11.3%; OR 1.37; 95% 0.79–2.38; *p* = 0.26; *I* ^2^ = 0%; Figure 4) were not significantly seen in the spironolactone group, as compared to the placebo group.

In a RCT with participants of both sexes, the efficacy of spironolactone (50 mg/day, including 27 women) was observed over a period of 3 months in a placebo‐controlled double‐blinded method [10]. Out of 30 participants in the spironolactone group, 24 (80%) patients had a response to spironolactone in the improvement of acne vulgaris. Among the 25 patients in the placebo group, 2 (8%) patients had improved acne vulgaris compared with spironolactone; distinctions in data were unavailable for women. Reversible menstrual irregularities were observed in 13 (48.1%) women out of 27 receiving spironolactone in this trial [10].

Due to high heterogeneity, a leave‐one‐out sensitivity analysis was performed for subjective assessment by iteratively removing one study at a time to ensure the results were not dependent on a single study. Overall, results showed a substantial change from the pooled analysis after sequential removal of each study, going from statistically nonsignificant to statistically significant. Removal of the latest study included in this meta‐analysis (SAFA 2023) yielded the heterogeneity to drop from *I* ^2^ = 85% to *I* ^2^ = 0% [6]. The sensitivity analysis results are described in Figures [Link], [Link], [Link].

TSA performed for objective assessment, demonstrated that the required information size was reached, suggesting no further trials are needed to validate the above results (Figure S6). Furthermore, it also showed that the position of the cumulative *Z*‐curve crossed beyond the conventional threshold and monitoring boundary into the area of benefit, favoring spironolactone over placebo.

### 3.3 Quality Assessment

Overall, two studies were classified as being high risk due to missing outcome data [8, 9]. Two were found with some concerns considering missing outcome data, respectively, and the randomization process [7, 10], while the most recent study was assessed as low risk of bias [6] (Figure S7).

## 4 Discussion

In this systematic review and meta‐analysis of 5 RCTs involving 563 patients, we compared the efficacy of spironolactone with placebo in the treatment of acne vulgaris in women. The main findings include: (1) a significant improvement in acne severity, as measured by objective assessments (OR 6.59; 95% CI: 3.50–12.43; *p* < 0.00001; *I* ^2^ = 0%); (2) no significant difference between spironolactone and placebo in subjective assessments of acne improvement (OR 5.22; 95% CI: 0.62–44.25; *p* = 0.13; *I* ^2^ = 85%), although sensitivity analysis indicated that high heterogeneity could be reduced by removing one study; (3) a lack of significant increase in adverse effects, including menstrual irregularities and breast enlargement, in the spironolactone group; and (4) TSA confirmed that the required information size had been reached, suggesting no further trials are needed to validate these findings.

In this meta‐analysis, spironolactone appears to offer a significant clinical benefit for women with acne, with no substantial increase in adverse effects, supporting its use as an effective treatment option. This significantly supports the 2024 AAD guidelines for acne, indicating that spironolactone can be considered based on individual patient factors, such as acne severity, treatment tolerance, patient preferences, and physician's judgment [3]. However, these guidelines were built based on a moderate level of evidence [3]. The TSA performed on the included studies supported these results, favoring spironolactone over placebo, and indicated the robustness of our results, showing that no more trials are needed to confirm our findings.

As aforementioned, leave‐one‐out analysis of acne improvement, based on subjective assessment, showed that the heterogeneity emerged from SAFA trial and resulted in a statistically significant difference, favoring spironolactone [6]. This can be attributed to the discrepancy in the methodology between older studies [9, 10] and the recent SAFA trial [6]. Older studies often followed protocols that, while appropriate for their time, lacked the rigorous design and standardized practices now considered essential. This includes less robust randomization techniques, smaller sample sizes, and outdated statistical methods, which likely contributed to the discrepancies in outcomes when compared to more recent studies. The usage of the participant's global assessment success score in SAFA trial, compared to its absence in the two older studies, may explain the differences in the results and support greater precision in SAFA, as it is based on a standardized scale.

Although it was not possible to perform a dosage‐based subgroup analysis in our meta‐analysis, a previous systematic review was performed based on cumulative low‐quality evidence, analyzing the dose of 200 mg/day of spironolactone for acne [6, 15, 16]. This dosage showed a highly significant improvement in inflamed lesions, while having more adverse events [6, 15, 16]. However, high doses of spironolactone (200 mg/day) are not usually prescribed for acne in clinical practice [6]. In our study, the most common dosage was 50 mg/day, and positive outcomes were still demonstrated.

Our findings suggest that adverse effects, such as menstrual irregularities and breast enlargement, were not statistically significant. This reflects the safety profile of spironolactone for acne. However, hyperkalemia was challenging to investigate in our meta‐analysis due to unreported data. A systematic review published in 2017, as well as one multicenter study, showed that it is unnecessary to monitor potassium levels, considering that the resultant hyperkalemia in the spironolactone group was mild and clinically insignificant [15, 17]. Even so, a recent cohort study on 32,234 patients showed that potassium level tests are usually ordered in patients taking spironolactone for acne [18]. It addresses the importance of investigating the best timing to address potassium level monitoring in clinical settings, as this was inconsistent among the patients included in the cohort [18]. It is suggested by AAD guidelines that potassium monitoring is not necessary in healthy individuals, and it should only be monitored if there are any potential risk factors, such as preexisting comorbidities and concomitant drug use that increase the risk of hyperkalemia [3]. Therefore, we suggest conducting future trials that include this endpoint in patients with risk factors to determine the best monitoring plan for these patients taking spironolactone.

Of note, several studies were conducted using the topical form of spironolactone, known as topical clascoterone, considering that it has fewer adverse effects than systemic drugs. A recent systematic review of clinical trials showed that topical spironolactone results in better outcomes than many other first‐line treatments, with fewer adverse effects [19]. A network meta‐analysis showed that clascoterone yields good outcomes in reducing inflammatory and noninflammatory lesion count [16]. One RCT compared the efficacy and safety of topical spironolactone versus topical dapsone for acne [20]. It showed that the therapeutic response differed significantly between the groups, with the topical spironolactone group showing a statistically favorable outcome [20]. This supports our findings, given that topical and oral spironolactone share the same drug configuration and metabolism, especially since topical spironolactone has been associated with a burning sensation [20].

This meta‐analysis' findings carry several important implications for future research. Our results indicate that oral spironolactone is effective and safe for acne compared to placebo; thus, future trials can consider comparing the efficacy and safety of oral spironolactone with FDA‐approved treatments for acne, such as retinoids, benzyl peroxide, topical dapsone, and antibiotics, to determine the best option in the therapeutic plan for acne in females. As shown in a recent RCT, oral spironolactone was 1.37 times and 2.87 times more successful in acne management than doxycycline at 2 respective times [21]. Also, the patient's quality of life and drug tolerance were better in the spironolactone group [21]. This reflects the potential of oral spironolactone for acne improvement and encourages the conducting of more studies. Moreover, we suggest that more studies should be directed based on the different doses of oral spironolactone to standardize the most efficient dose with the least adverse events. As we demonstrated the efficacy of spironolactone in female patients, further trials can warrant sex‐specific efficacy and safety of spironolactone compared with standard acne treatment, taking precautions in men due to the lack of evidence supporting the robustness of spironolactone and its effectiveness in men [22]. Given the widespread clinical use of this medication, we recommend that it be incorporated into future acne treatment guidelines and considered for FDA approval. This would provide clinicians with evidence‐based support for its use and promote standardized care for patients.

Our study has limitations. Firstly, it was not feasible to analyze the adjusted ORs for the baseline characteristics, such as hormonal or topical treatment, age, or polycystic ovary syndrome (PCOS) status, due to unreported data; it was available in the SAFA trial and only for two outcomes: objective and subjective assessments. Secondly, it is important to note that 77% of the population in the SAFA trial (30% in the spironolactone group and 47% in the placebo group) had either a suspicion or diagnosis of PCOS [6]. This makes it difficult to attribute the observed effects of menstrual irregularities to either the underlying condition or the use of spironolactone. Thirdly, in the study by Goodfellow, ages of male and female participants was combined due to the limited number of participants of each sex, which prevented us from reporting the mean age of only females in Table 1 [8]. In the Mansurul study, baseline characteristics for grading acne were only provided for the subset of patients who adhered to the 3‐month treatment protocol. This reduces the generalizability of the acne grading results to the initial cohort and may overestimate the treatment efficacy by only considering those who followed the treatment as prescribed. Additionally, outcomes for male and female participants were not reported separately. This lack of stratification by gender makes it impossible to include its findings in our statistical analysis [10]. Fifth, while we reported the adverse effects that were commonly observed in the studies, other adverse effects were mentioned but were not frequently reported. However, these less commonly reported effects may not have been directly attributed to spironolactone use; therefore, future trials should evaluate these adverse events to evaluate the safety of spironolactone.

## 5 Conclusion

In conclusion, this systematic review and meta‐analysis based on available evidence suggests that spironolactone may improve acne vulgaris in women without a significant increase in adverse effects. TSA, which accounts for the risk of bias in included studies, indicates that further trials may not be necessary; however, the overall quality of the included studies is limited, which should be considered when interpreting these findings.

## Author Contributions

**Laura Ghanem:** conceptualization (lead), data curation (equal), formal analysis (equal), investigation (lead), methodology (lead), project administration (equal), validation (equal), visualization (equal), writing – original draft (equal); writing – review and editing (equal). **Najwaa Kirmani:** conceptualization (lead), data curation (equal), formal analysis (equal), investigation (lead), methodology (lead), project administration (equal), validation (equal), visualization (equal), writing – original draft (equal), writing – review and editing (equal). **Nathalia De León Fernández:** conceptualization (supporting), data curation (lead), formal analysis (equal), methodology (equal), validation (equal), visualization (equal), writing – original draft (equal), writing – review and editing (equal). **María Paula Palacios‐Ortiz:** data curation (lead), formal analysis (equal), methodology (equal), validation (equal), visualization (equal), writing – original draft (equal), writing – review and editing (equal). **Juan David Rodríguez‐Parra:** data curation (equal), formal analysis (equal), methodology (equal), validation (equal), visualization (equal), writing – original draft (equal), writing – review and editing (equal). **Corina A. Rusu:** data curation (supporting), formal analysis (supporting), investigation (equal), methodology (supporting), supervision (lead), validation (equal), visualization (equal), writing – review and editing (equal).

## Ethics Statement

The authors have nothing to report.

## Consent

The authors have nothing to report.

## Conflicts of Interest

The authors declare no conflicts of interest.

## Supporting information


**Appendix S1:** jocd70411‐sup‐0001‐AppendixS1.pdf.

**Figure S1:** The severity of acne vulgaris was significantly reduced in women taking spironolactone as compared to placebo, following the objective assessment. CI, confidence interval; IV, inverse variance; OR, odd ratio; SE, standard error.

**Figure S2:** The severity of acne vulgaris was nonsignificantly reduced in women taking spironolactone as compared to placebo, following the subjective assessment. CI, confidence interval; IV, inverse variance; OR, odd ratio; SE, standard error.

**Figure S3:** The leave‐one‐out analysis of Goodfellow et al. for the subjective assessment of acne improvement did not show a decrease in heterogeneity among the studies. CI, confidence interval; IV, inverse variance; OR, odd ratio; SE, standard error.

**Figure S4:** The leave‐one‐out analysis of Muhelmann et al. for the subjective assessment of acne improvement didn't show a decrease in heterogeneity among the studies. CI, confidence interval; IV, inverse variance; OR, odd ratio; SE, standard error.

**Figure S5:** The leave‐one‐out analysis of SAFA for the subjective assessment of acne improvement showed a decrease in heterogeneity among the studies to 0%. CI, confidence interval; IV, inverse variance; OR, odd ratio; SE, standard error.

**Figure S6:** Trial Sequential Analysis (TSA) based on objective assessment, demonstrating that the required information size was reached. The cumulative *Z*‐curve crosses the conventional threshold and monitoring boundary, indicating a significant benefit of spironolactone over placebo and suggesting that further trials may not be necessary to confirm these findings.

**Figure S7:** Quality assessment of the RCTs per Cochrane RoB‐2.

## Data Availability

All data analyzed in this meta‐analysis were obtained from studies retrieved from PubMed (https://pubmed.ncbi.nlm.nih.gov), Embase (www.embase.com), and the Cochrane Library (www.cochranelibrary.com). All supporting data from the included studies are publicly available and are cited within the manuscript.
