# Supplementary material for: Efficacy and Safety of Oral Spironolactone for Women With Acne Vulgaris: A Systematic Review and Meta‐Analysis of Randomized Placebo‐Controlled Trials With Trial Sequential Analysis
Source: J Cosmet Dermatol. 2025 Aug 18;24(8):e70411. doi: 10.1111/jocd.70411 (PMC12359290; doi:10.1111/jocd.70411)
Supplement: Supplementary file 1 — Appendix S1: jocd70411‐sup‐0001‐AppendixS1.pdf. [file JOCD-24-e70411-s004.pdf]

Search Strategy

- **PubMed**

(Spironolactone OR "Spironolactone"[Mesh] OR Aldactone OR Verospiron OR Spirolone OR “SC 9420” OR Spironolactonum OR “mineralocorticoid receptor antagonist” OR "Mineralocorticoid Receptor Antagonists"[Mesh] OR “aldosterone antagonist”) AND (acne OR "Acne Vulgaris"[Mesh] OR comedones OR seborrhea OR “Propionibacterium acnes” OR “Cutibacterium acnes”) AND ("randomized controlled trial"[pt] OR "controlled clinical trial"[pt] OR randomized[tiab] OR placebo[tiab] OR "drug therapy"[sh] OR randomly[tiab] OR trial[tiab] OR groups[tiab])

- **Embase**

(Spironolactone OR Aldactone OR Verospiron OR Spirolone OR “SC 9420” OR Spironolactonum OR “mineralocorticoid receptor antagonist” OR “aldosterone antagonist”) AND (acne OR "Acne Vulgaris" OR comedones OR seborrhea OR “Propionibacterium acnes” OR “Cutibacterium acnes”) AND ('randomized controlled trial'/exp OR 'controlled clinical trial'/exp OR randomized:ti,ab OR placebo:ti,ab OR 'drug therapy'/exp OR randomly:ti,ab OR trial:ti,ab OR groups:ti,ab)

- **Cochrane**

(Spironolactone OR Aldactone OR Verospiron OR Spirolone OR “SC 9420” OR Spironolactonum OR “mineralocorticoid receptor antagonist” OR “aldosterone antagonist”) AND (acne OR "acne vulgaris” OR comedones OR seborrhea OR “Propionibacterium acnes” OR “Cutibacterium acnes”)
