# Supplementary figures and images for: Efficacy and Safety of Oral Spironolactone for Women With Acne Vulgaris: A Systematic Review and Meta‐Analysis of Randomized Placebo‐Controlled Trials With Trial Sequential Analysis
Source: J Cosmet Dermatol. 2025 Aug 18;24(8):e70411. doi: 10.1111/jocd.70411 (PMC12359290; doi:10.1111/jocd.70411)

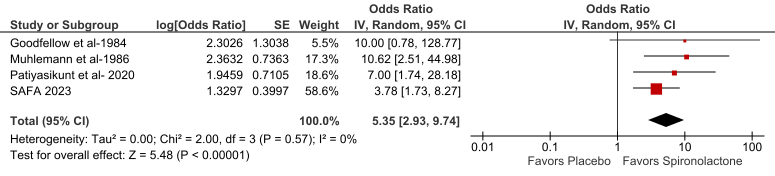

Supplement: Supplementary file 2 — Figure S1: The severity of acne vulgaris was significantly reduced in women taking spironolactone as compared to placebo, following the objective assessment. CI, confidence interval; IV, inverse variance; OR, odd ratio; SE, standard error. [file JOCD-24-e70411-s002.png]

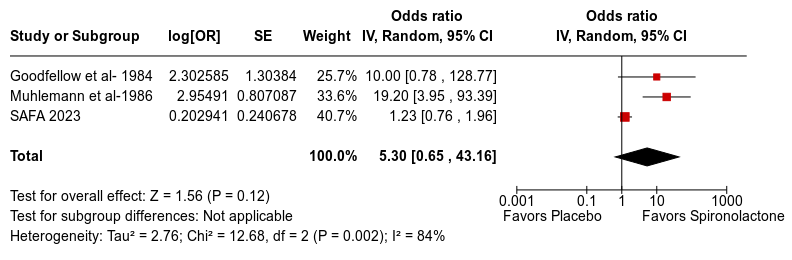

Supplement: Supplementary file 3 — Figure S2: The severity of acne vulgaris was nonsignificantly reduced in women taking spironolactone as compared to placebo, following the subjective assessment. CI, confidence interval; IV, inverse variance; OR, odd ratio; SE, standard error. [file JOCD-24-e70411-s005.png]

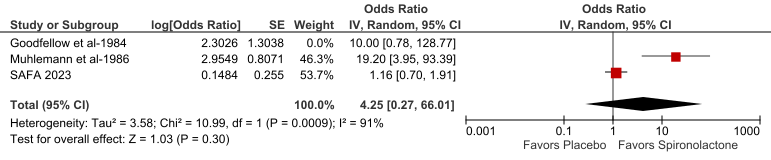

Supplement: Supplementary file 4 — Figure S3: The leave‐one‐out analysis of Goodfellow et al. for the subjective assessment of acne improvement did not show a decrease in heterogeneity among the studies. CI, confidence interval; IV, inverse variance; OR, odd ratio; SE, standard error. [file JOCD-24-e70411-s007.png]

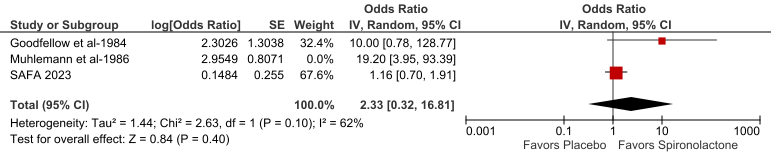

Supplement: Supplementary file 5 — Figure S4: The leave‐one‐out analysis of Muhelmann et al. for the subjective assessment of acne improvement didn't show a decrease in heterogeneity among the studies. CI, confidence interval; IV, inverse variance; OR, odd ratio; SE, standard error. [file JOCD-24-e70411-s008.png]

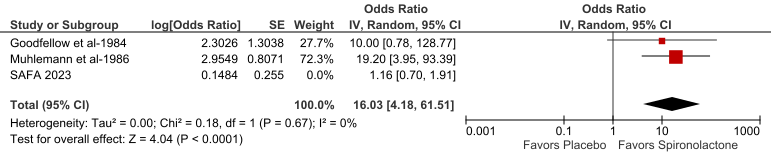

Supplement: Supplementary file 6 — Figure S5: The leave‐one‐out analysis of SAFA for the subjective assessment of acne improvement showed a decrease in heterogeneity among the studies to 0%. CI, confidence interval; IV, inverse variance; OR, odd ratio; SE, standard error. [file JOCD-24-e70411-s003.png]

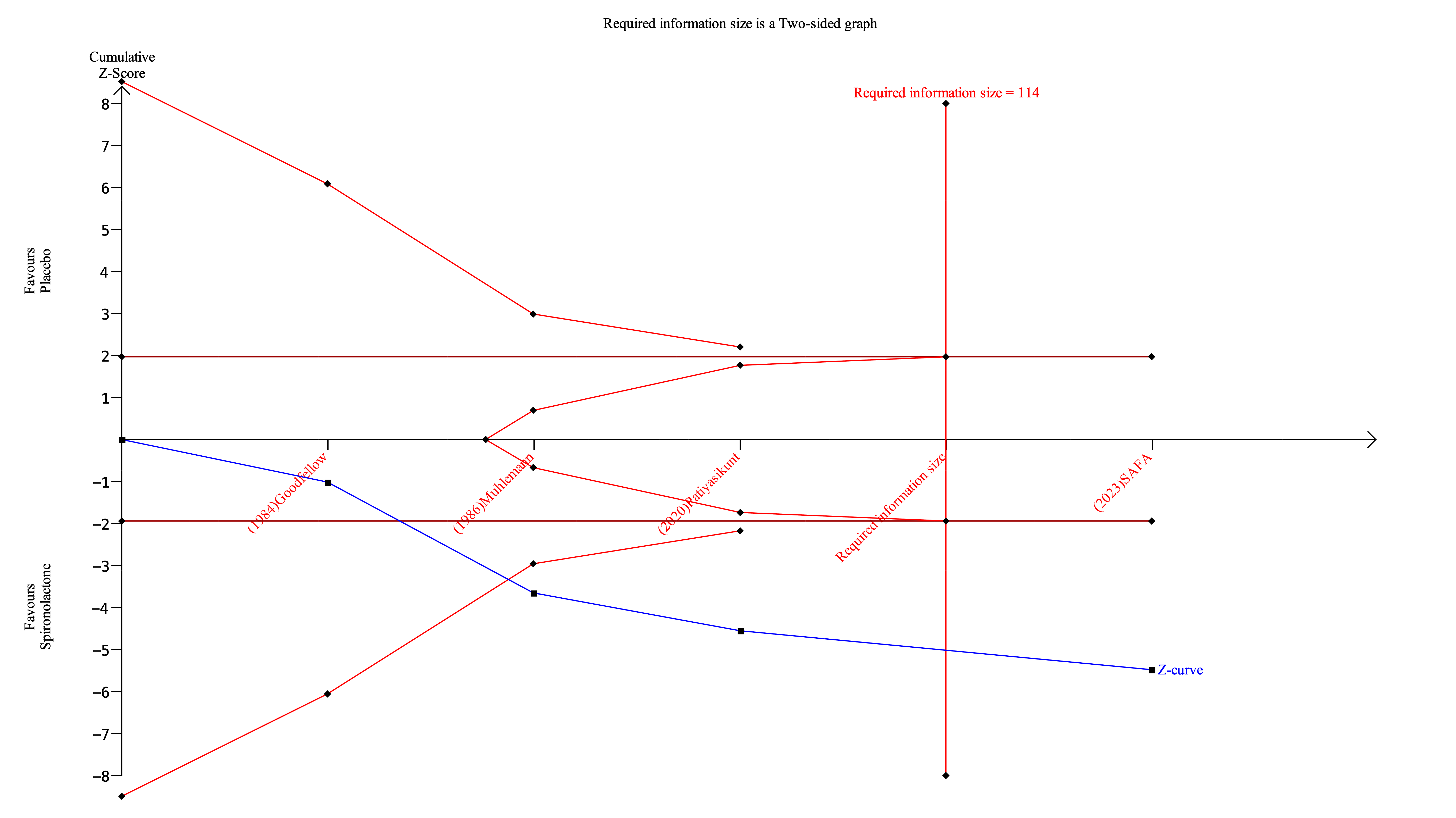

Supplement: Supplementary file 7 — Figure S6: Trial Sequential Analysis (TSA) based on objective assessment, demonstrating that the required information size was reached. The cumulative Z‐curve crosses the conventional threshold and monitoring boundary, indicating a significant benefit of spironolactone over placebo and suggesting that further trials may not be necessary to confirm these findings. [file JOCD-24-e70411-s001.png]

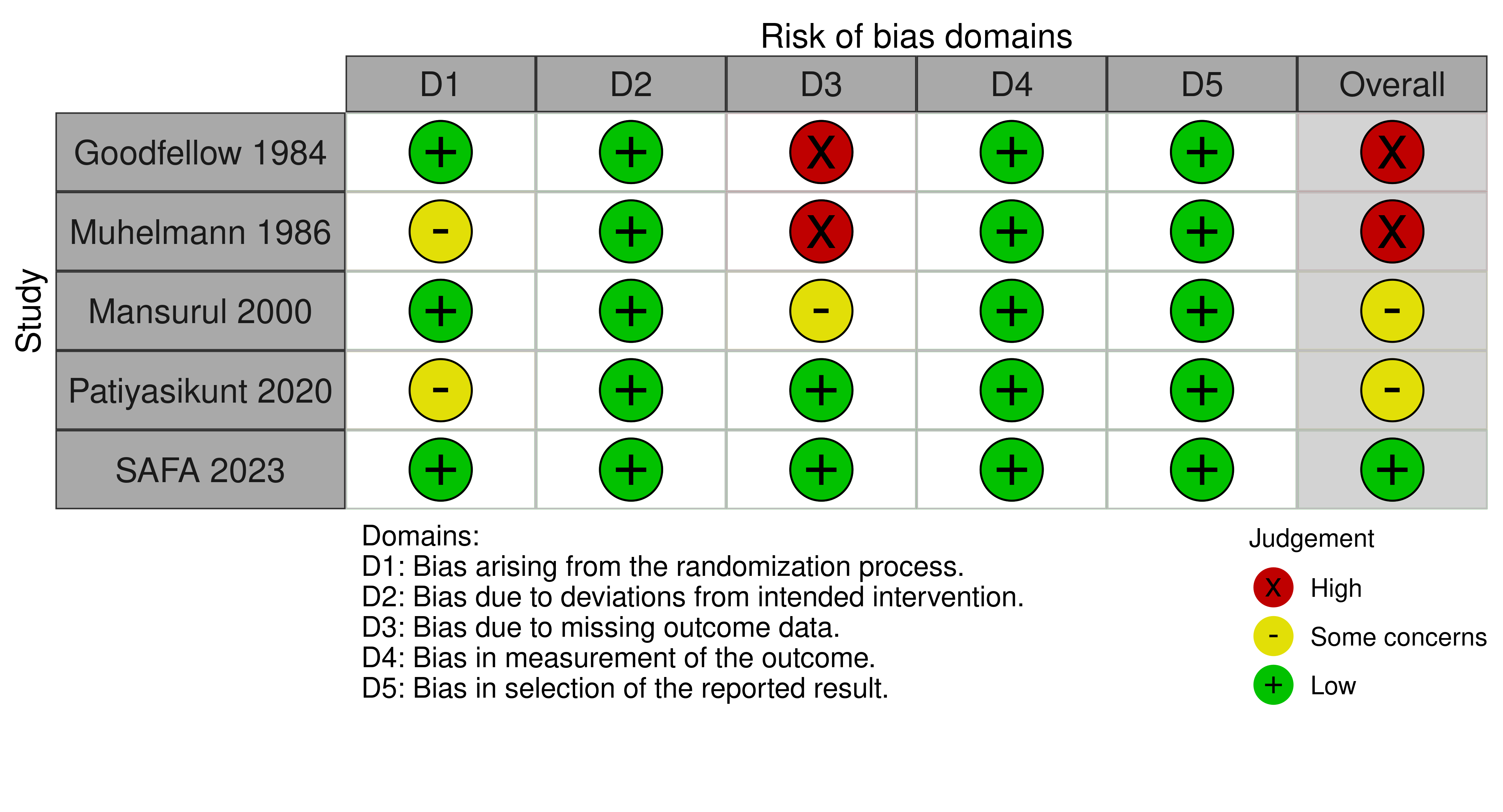

Supplement: Supplementary file 8 — Figure S7: Quality assessment of the RCTs per Cochrane RoB‐2. [file JOCD-24-e70411-s006.png]
